# Management of Root Fracture: A Novel, Noninvasive Treatment Approach

**DOI:** 10.1155/2013/653261

**Published:** 2013-10-27

**Authors:** M. S. Rangareddy, Archana Daga, Y. Vishnu Vardhan, M. Daneswari

**Affiliations:** ^1^Panineeya Institute of Dental Sciences, Hyderabad, Andhra Pradesh 500060, India; ^2^Mamtha Dental College, Khammam, Andhra Pradesh 500060, India

## Abstract

Traumatic injuries to teeth account for approximately 25% of dental conditions where a patient seeks dentist for emergency treatment. Radicular fractures are one such entity which is very challenging to address due to various complications like periodontal communication, increased mobility, and continued pulpal infection leading to necrosis. Radicular fractures in the middle third have long been considered teeth of salvage due to their unfavourable fracture pattern. During the recent years introduction of biomimetic materials has opened the horizon for saving these teeth. In the present case report a novel approach to the management of radicular fractures in the middle third has been presented.

## 1. Introduction

Traumatic injuries to a tooth range in severity from a simple enamel infraction to a complete exarticulation of tooth also known as avulsion. These injuries account for being the third most common cause for tooth loss where the patient seeks the dentist for emergency treatment [1]. The clinically challenging cases of root fractures due to their complex management involving interdisciplinary/multidisciplinary treatment approach are of particular interest to the clinician [2]. Root fractures are a rare entity where their frequency in permanent teeth is only from 0.5% to 7% and in deciduous teeth is from 2% to 4% [1]. Root fractures occur mainly in the maxillary centrals (68%) and maxillary laterals (27%) mainly due to frontal impact with a rare involvement of only 5% in mandibular incisors [3].

 These horizontal fractures are mainly divided on the location of fragment into cervical, middle, and coronal third fractures. Among these entities middle third fractures are relatively more common. Middle third fractures are generally transverse to oblique and may be single or multiple, complete or incomplete [4]. Treatment approach for these fractures is complex involving prognostic considerations like degree of displacement of fracture fragment, patient's age, stage of root growth, mobility of the coronal fragment, and diastasis of the fragments [1].

Endodontic treatment is required in these situations as they lead to pulp necrosis. The various treatment approaches available include either root canal therapy of both fracture fragments [5]; this may be indicated in fracture cases when the segments are not separated and where complete dryness of canal can be obtained. Another treatment approach consists of root canal treatment of the coronal segment only, if this segment shows no mobility [6]. The use of an intraradicular splint in the form of post systems has also been recommended. In the present case reports a novel approach to the management of root fractures is carried out with the use of MTA as intraradicular splint along with aesthetic management with direct and indirect composites. 

## 2. Case ****1

**Figure 1:**
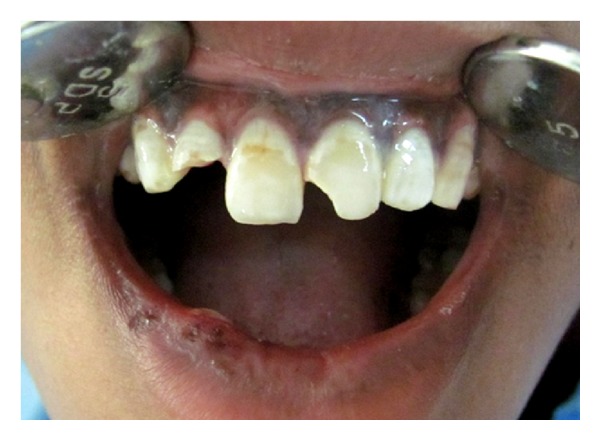
Diagnostic photo.

An 18-year-old female reported to the Department of Conservative and Endodontics, Panineeya Institute of Dental Sciences, Hyderabad, India, with a chief complaint of broken upper front tooth and mobile upper front tooth for 2 days. On expanding the history, the patient elicited trauma due to road traffic accident two days back. Clinical extraoral examination revealed lacerations on the lower lip. Intraoral examination revealed grade II mobility i.r.t 11 and complicated crown fracture i.r.t 12 (Figure 1).

**Figure 2:**
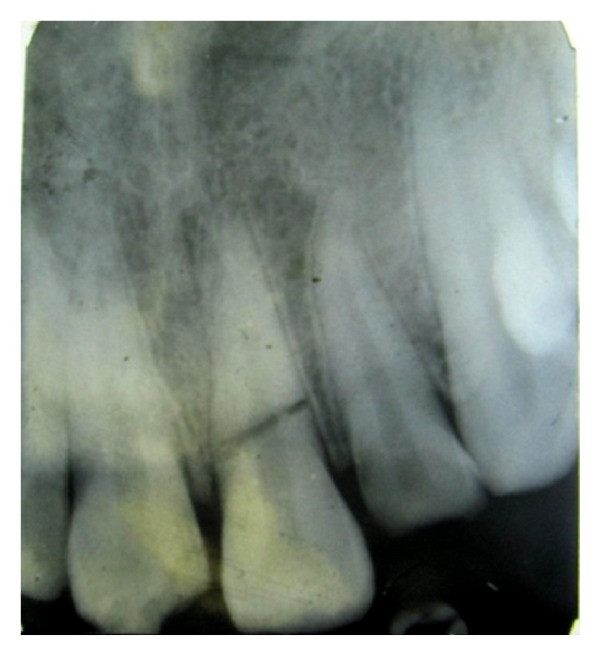
Diagnostic radiograph.

On investigating the intraoral periapical radiograph (Figure 2), a horizontal fracture line was seen i.r.t 11 at the junction of coronal and middle third with minimal displacement of fracture fragments. So the final diagnosis was established as Ellis Class VI fracture i.r.t 11, Ellis Class III fracture i.r.t 12, Ellis Class I fracture i.r.t 21, with moderate dental fluorosis. 

Taking into consideration the prognostic factors regarding root fracture i.r.t 11, patient was clearly informed about the various treatment options available either to save the tooth or to send it for extraction. On obtaining the informed consent, a comprehensive treatment plan was devised for the patient which consisted of managing the root fracture i.r.t 11 with MTA as a splint material, post and core rehabilitation for tooth number 12, along with direct and indirect composite restorations. 

**Figure 3:**
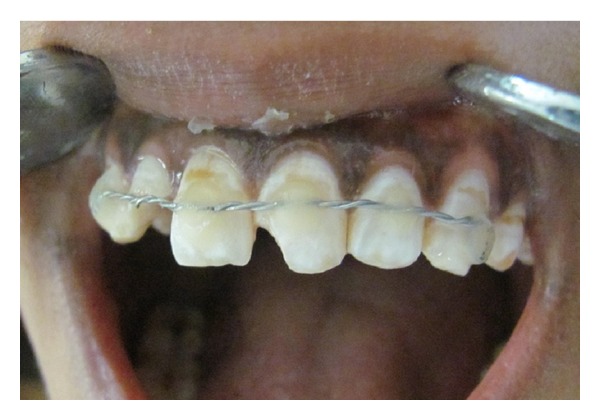
Splinting.

Initial treatment began with splinting the teeth using orthodontic braided wire and flowable composite as a rigid splint for a period of 6 weeks according to Andreason's protocol (Figure 3).

**Figure 4:**
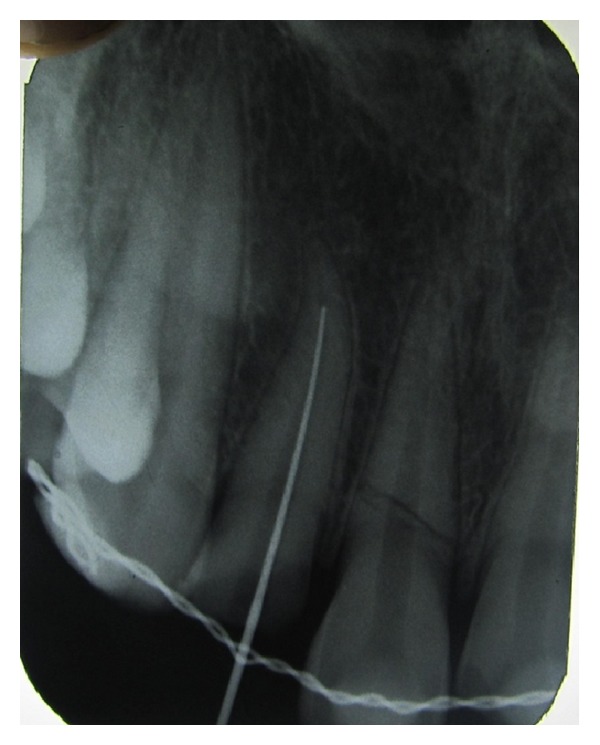
Working length 12.

**Figure 5:**
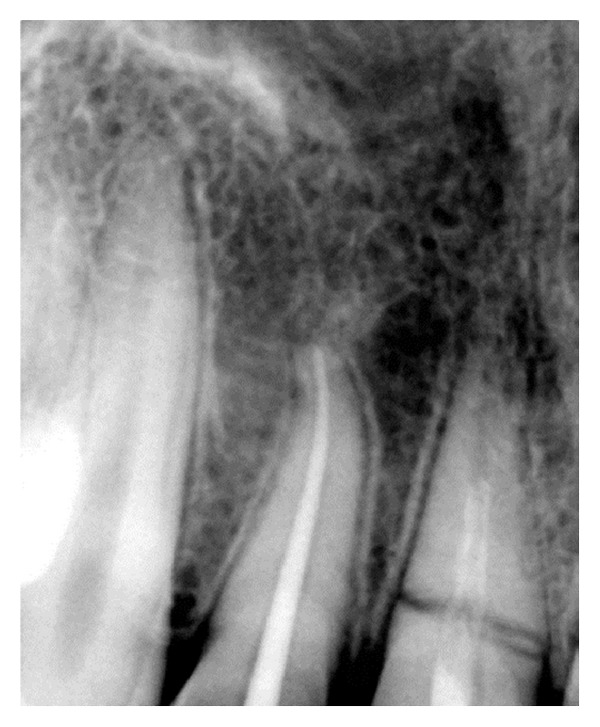
Master cone 12.

**Figure 6:**
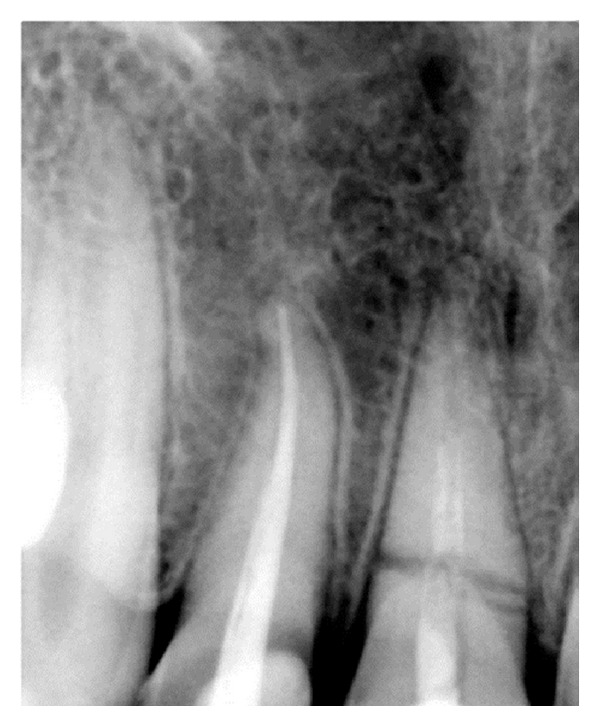
Obturation 12.

**Figure 7:**
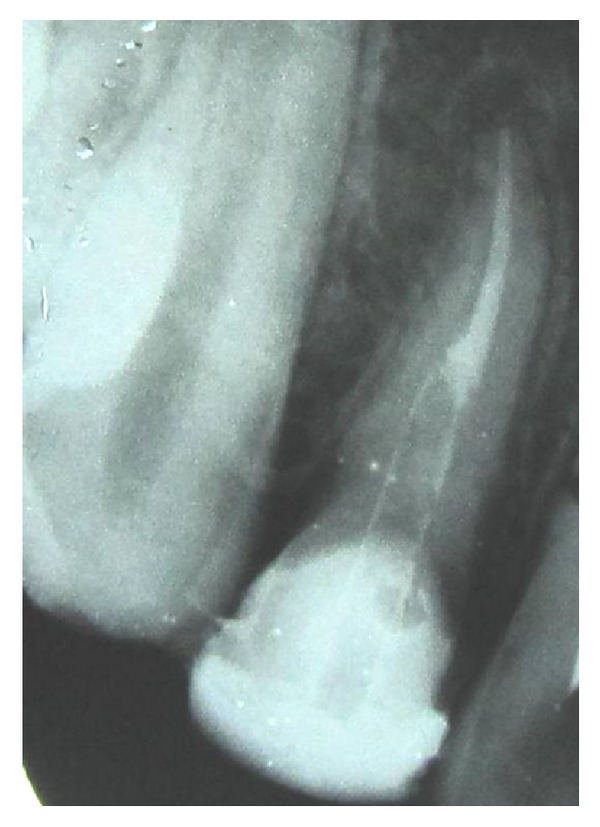
Post and core 12.

**Figure 8:**
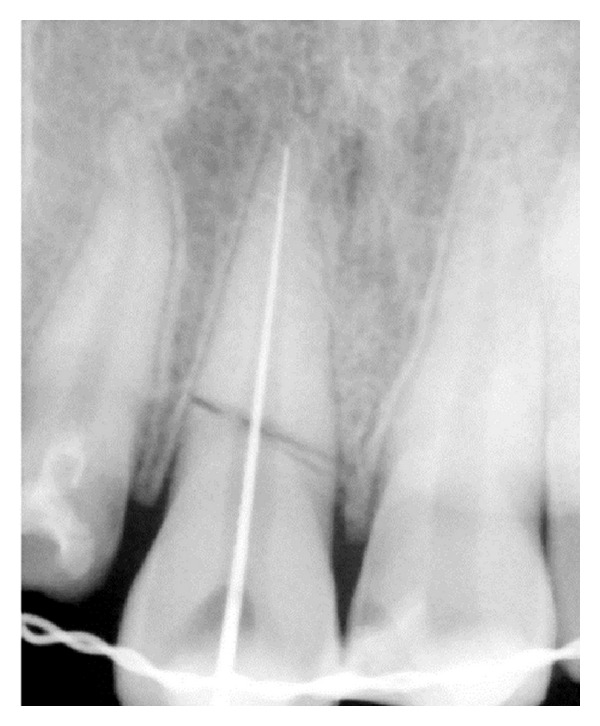
Working length 11.

**Figure 9:**
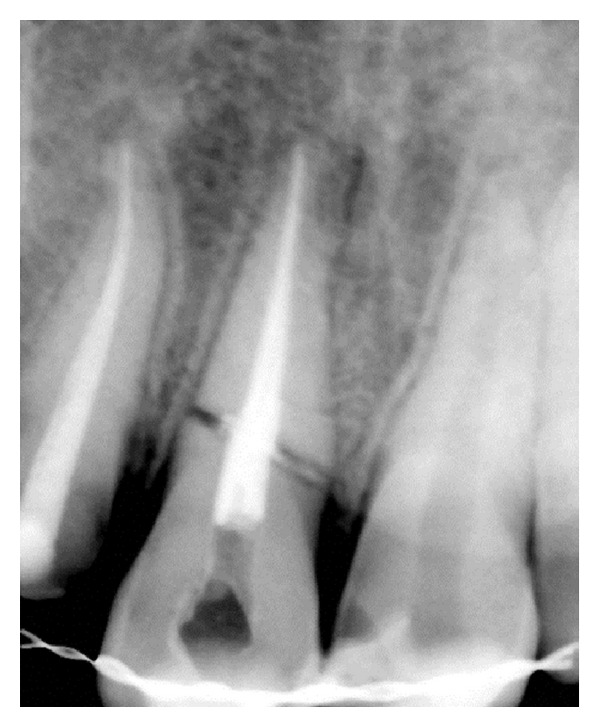
Obturation 11.

**Figure 10:**
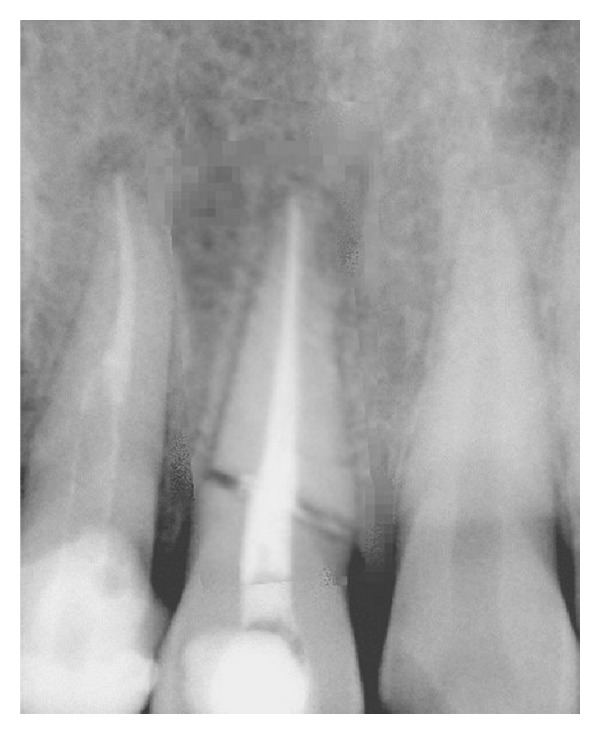
MTA placement.

**Figure 11:**
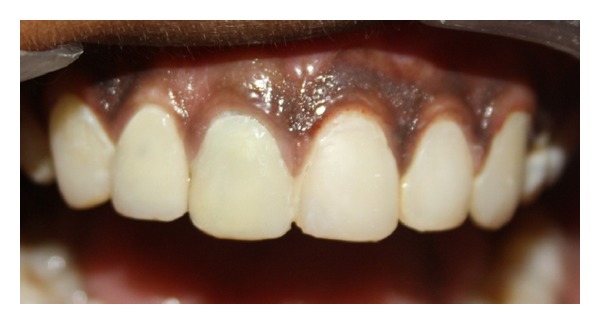
Direct and indirect composite restorations.

Endodontic treatment was initiated i.r.t 11 and 12; after access opening of 12, cleaning and shaping were carried out with hand K-files using step back technique, followed by obturation by using resin sealer (AH Plus; Dentsply; lot number 1209000390, Germany) and lateral condensation method. Later post space preparation was done to receive fibre-post (Quartzix Added Posts; number 2, Landy, Swiss Dental Products Of Distinction) (Figures 4, 5, 6, and 7). The final coronal restoration was planned as indirect composite crown (Adoro Indirect Composite, Ivoclar Vivadent) (Figure 11). For tooth number 11, endodontic treatment was carried out after two weeks confirming pulp necrosis as a result of checking the pulp vitality. After access preparation, number 15 K File was used to negotiate through the fracture fragment (Figure 8). After initial cleaning and shaping using hand files to ensure complete asepsis, two weeks of intracanal calcium hydroxide paste were given (ApexCal, Ivoclar Vivadent). When we could ensure complete dryness of the canal without any bleeding, obturation was carried out with Zinc Oxide Eugenol Sealer (Deepak Enterprises, Mumbai) and lateral condensation of gutta-percha through the complete root. Following this, gutta-percha is carefully removed just below the fracture extension (Figure 9). Later, MTA (Proroot, Dentsply, Germany) is densely packed with hand pluggers (Figure 10) through the fracture fragment and access cavity sealed with flowable composite. Esthetic management for this tooth consisted of a conservative approach of giving indirect composite veneer (Adoro Indirect Composite, Ivoclar Vivadent) (Figure 11).

**Figure 12:**
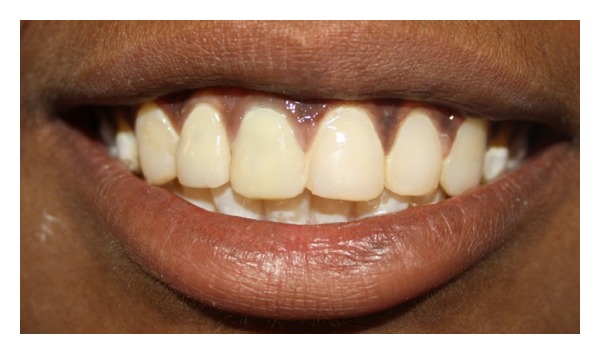
Six-month recall.

To address fluorosis, direct composite veneers were given i.r.t 13, 21, 22, and 23 which gave a uniform shade to the patient. Immediate postop evaluation revealed reduction in tooth mobility i.r.t 11 and followup of 6 months did not reveal any periapical changes with the patient remaining asymptomatic (Figure 12).

## 3. Case ****2

**Figure 13:**
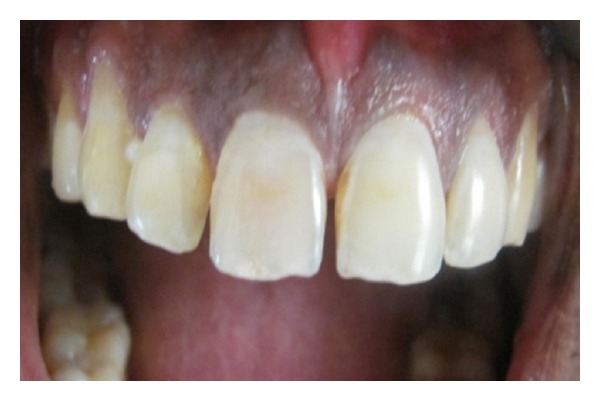
Diagnostic picture.

**Figure 14:**
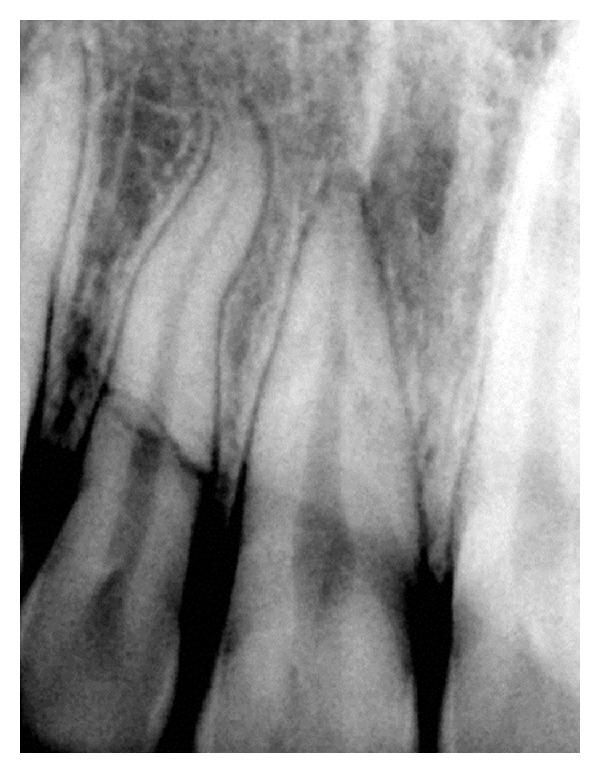
Diagnostic radiograph.

 A 26-year-old male patient reported to our department with a chief complaint of mobile upper front tooth following a fist injury. On clinical and radiographic examination a diagnosis of Ellis Class VI fracture was established i.r.t 12 (Figures 13 and 14).

**Figure 15:**
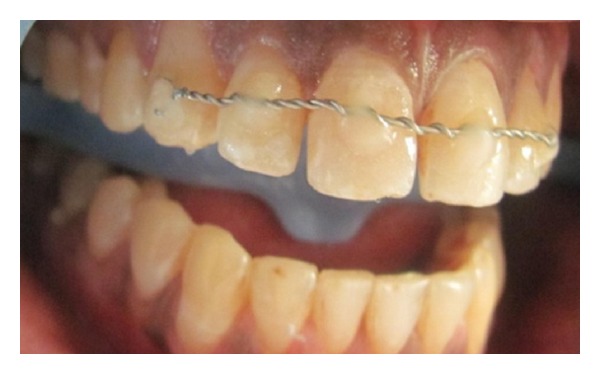
Splinting of teeth.

 The treatment protocol was similar to that of Case 1, where rigid splinting was carried out using orthodontic braided wire and flowable composite for a period of 6 weeks (Figure 15). 

**Figure 16:**
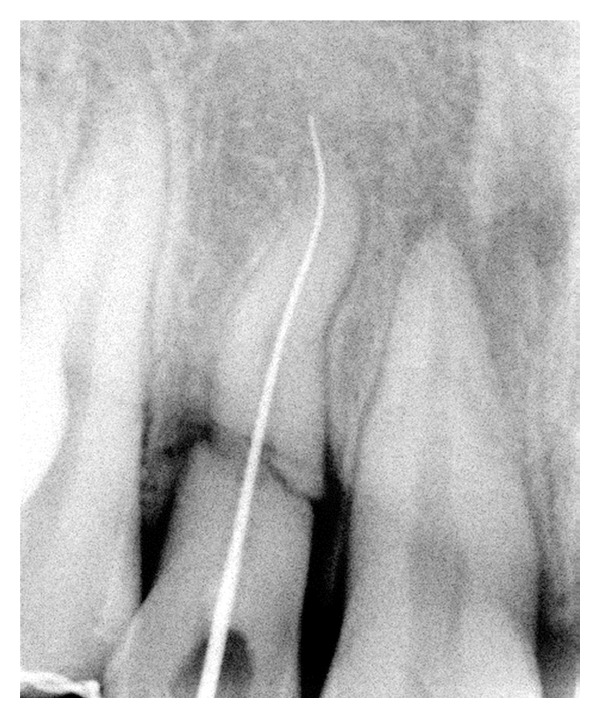
Working length 12.

**Figure 17:**
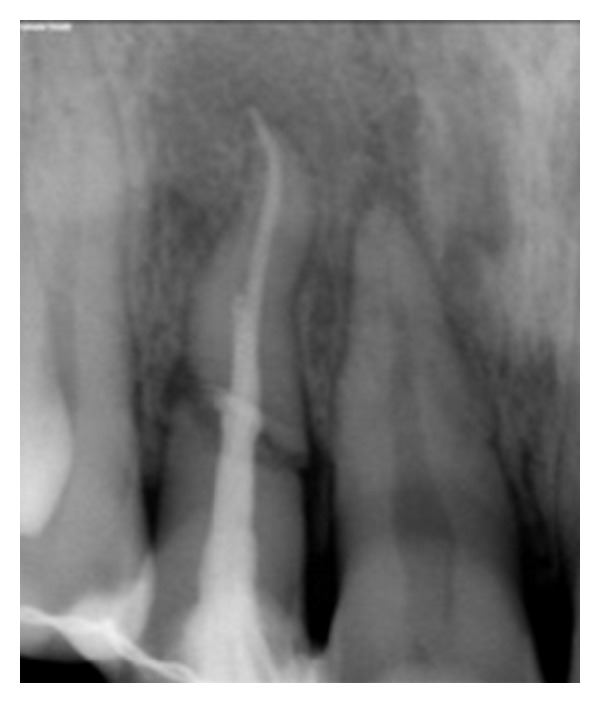
Obturation 12.

**Figure 18:**
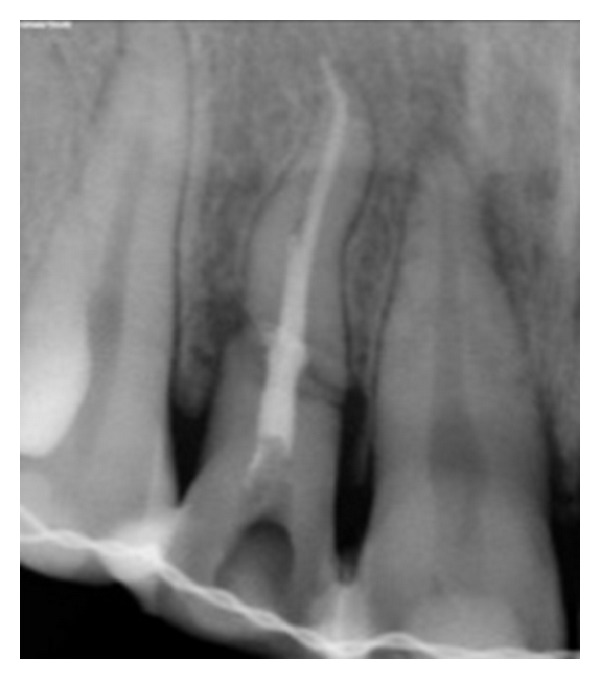
MTA placement.

 Endodontic treatment began two weeks after splinting of teeth, where tooth vitality was checked indicating delayed response, access preparation and pulp extirpation was carried out, and working length was determined (Figure 16). Due to the bayonet configuration of the root, complete instrumentation was done using hand Ni-Ti files by step back technique; following this, complete obturation (Figure 17) was carried out using ZOE sealer and lateral condensation of gutta-percha. And similar intraradicular splinting was carried out like in Case 1 using white MTA (Proroot, Dentsply, Germany) (Figure 18).

**Figure 19:**
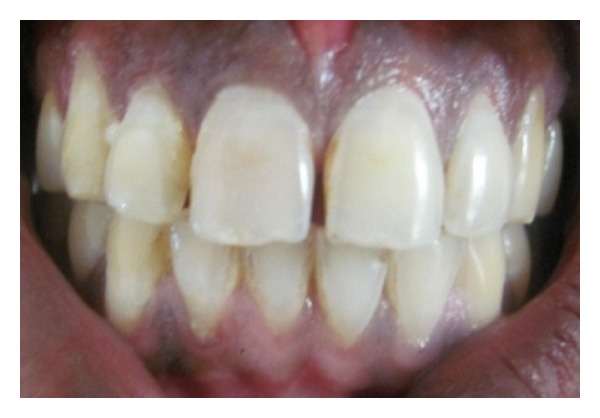
Splint removal and followup.

 Immediate postop evaluation revealed complete resolution of tooth mobility to physiological limits and remaining teeth showed a vital response (Figure 19).

## 4. Discussion

 Management of horizontal middle third root fractures is challenging to an endodontist due to the association of pulpal and periodontal components. Hence the ultimate goal to preserve the natural dentition encourages the possibilities of new horizons to manage these critical situations. The influence of “preinjury and injury factors” on the healing of intraalveolar root fractures was carried out in a study, whose authors found that the age of the patients, the stage of root growth, mobility of the coronal fragment, dislocation of the coronal fragment, and fragment diastasis exerted the greatest influence on healing at the fracture line and on the occurrence of pulpal necrosis [1]. A recent study by Cvek et al. concluded that 20% of teeth with root fracture lead to pulp necrosis indicating the importance of early endodontic intervention in these patients [7]. 

In both mentioned cases, the treatment plan was decided based on the level of fracture fragment to the crest of the alveolar bone. As the fragments had the fracture line extending closer to the crest, complete endodontic treatment was considered involving both the coronal and apical fragments. Endodontic treatment was carried out as described in the guidelines given by the International Association of Dental Traumatology (IADT) [6]. Nevertheless, vitality was checked two weeks after the trauma and both cases showed delayed response suggestive of pulp necrosis. Cvek et al. reported in their study on the exclusion of the apical fragment due to problems of infection as in case of root fractures [8]. But by doing so there would be a compromise in the crown root ratio of the teeth; hence negotiation and obturation of fracture fragment were also considered. Hand files were used in both cases to preserve maximum intraradicular dentin. 

 A novel approach of using MTA as a splint material over the fracture fragments was utilised due to its various properties of being osseoinductive, which would result in formation of a hard tissue around the fracture site high pH contributes to its bactericidal effects which creates a sterile environment around the fracture site. And it is biocompatible which explains that minor leakage in Case 1 did not contribute to any periodontal changes [9], and it has a hard set stabilization which would contribute to it acting as an intraradicular splint. Furthermore due to its excellent biocompatibility leakage of MTA helps in the healing of periodontal apparatus by forming normal architecture [10]. 

In Case 1 esthetic rehabilitation was carried out by direct and indirect composites. Indirect composites were used in this case due to their conservation of tooth structure and improved esthetics when compared to direct composites [11].

Short-term followup of both cases have shown promising results of this novel approach. Regular followup along with more clinical trials can confirm this treatment modality for fractured roots.

## 5. Conclusion

 Middle third fractures have long been considered to have a poor prognosis because of lack of understanding the biologic concept of such fracture along with insufficient knowledge to manage these situations. 

In recent years introduction and availability of biocompatible materials like MTA have opened the horizons for the clinicians to put forth varied treatment options in the management of mid-root fractures.
